# Participants' perspective on a COVID-19 online vocal group stimulation for people with Parkinson's disease

**DOI:** 10.3389/fresc.2022.951426

**Published:** 2022-08-02

**Authors:** Marie-Christine Hallé, Charline Delorme, Édith Coulombe, Ouswa Rekik, Ingrid Verduyckt

**Affiliations:** ^1^Laboratoire IV, École d'orthophonie et d'audiologie, Université de Montréal, Montréal, QC, Canada; ^2^Centre de Recherche Interdisciplinaire en Réadaptation (CRIR) du Montréal Métropolitain, Montréal, QC, Canada

**Keywords:** Parkinson's disease, COVID-19, communication, speech, voice, intervention, telehealth, accessibility

## Abstract

The COVID-19 related confinement and social distancing had negative consequences on the health of individuals living with Parkinson's Disease (PD). In collaboration with a non-profit organization, we developed and implemented a daily online vocal stimulation group named “Musculation de la Voix” (MdlV) in April 2020. To better understand the potential of MdlV to diversify existing services available to people with PD experiencing vocal symptoms, this study aimed to explore participants' experience and perception of MdlV in terms of participation, motivations, feelings, perceived changes, and appreciation. The 45 individuals who registered to the Summer 2020 Semester of MdlV were invited to complete an online ten-question survey. Responses to the four close-ended questions were analyzed using descriptive statistics while statements provided in response to the six open-ended questions were subjected to an inductive qualitative content analysis. Thirty seven participants completed the survey. Results revealed that the sample of respondents was mostly constituted of individuals who were engaged in this activity since its very beginning (62,2%), participated daily (59,5%), intended to keep participating in the activity (97,3%), and had never received speech-language therapy (SLT) services before (72,97%). The qualitative analysis yielded one theme relating to prior services: “Previous SLT services are variable and perceived as beneficial but with limitations,” and three themes pertaining to MdlV: “Seeking improvement and support as initial motivations to engage in MdlV,” “Unanticipated benefits and desired gains catalyzing motivation to participate in MdlV,” and “Perceived limitations of MdlV and persisting needs.” Our study participants' engagement and motivation toward MdlV as well as the benefits they perceived in relation to this activity suggest that an online vocal stimulation group may be a promising complement to currently limited SLT services. As limitations and persisting needs were also identified, future studies are required to elucidate what aspect of MdlV works, for whom and how.

## Introduction

The COVID-19 pandemic and the public health measures implemented to reduce the spread of the virus, including social distancing and confinement, have caused social isolation and breakdown of services in healthcare institutions and community. Such disruptions were most likely to affect individuals already at risk of social isolation and for whom healthcare and community services were essential to support their physical and mental health. Among these people are those with Parkinson's disease (PD), the second most common neurodegenerative disorder in Canada and in the United States (1, 2). The loss of dopamine production characterizing this disease is associated with a variety of symptoms including tremors, rigid and slow movements, balance problems, speech and swallowing disorders, loss of facial expressions, depression, anxiety and cognitive impairment (2, 3) that bring about challenges such as a decreased ability to perform activities of daily living and a change in self-image that can cause social anxiety and isolation (4).

Adapting to the myriad of symptoms caused by PD requires some changes in one's lifestyle and habits (5). This adaptation process was likely to be disrupted by the new reality imposed by the COVID-19 pandemic. For many individuals living with PD, the confinement due to COVID-19 pandemic was synonym of decreased levels of physical activity, increased anxiety, worsened cognitive abilities, diminution of quality of sleep, deterioration in psychological health and aggravation of motor symptoms (6–9). Women living alone were particularly likely to experience negative changes in their health and symptoms (6, 7).

To mitigate the negative consequences of COVID-19 in the daily life of people with PD, Parkinson Québec, a non-profit organization in the province of Quebec, Canada, called upon our team of researchers in speech-language therapy (SLT) to design a solution that would allow its members to break the social isolation. Being aware of the potential negative consequences of limited social interaction on the vocal function of people with PD, our team designed a two in one activity that would provide participants with the opportunity to break the social isolation while regularly practicing their voice despite confinement and social distancing. More specifically, principles of neuroplasticity such as “Use It or Lose It” and “Use It and Improve It” (10), which suggest that neural circuits unsolicited in task performance for an extended period of time begin to degrade whereas the use of these circuits can lead to the improvement of the underlying functions, led our team to propose online daily voice stimulation sessions entitled “Musculation de la Voix” (MdlV).

MdlV was designed as the equivalent of a daily walk, but for the voice. These 30 min virtual sessions, offered every weekday, are dedicated to voice workout and socialization with a group of participants with Parkinson's disease. The sessions are facilitated by SLT students as well as by co-author IV, the project coordinator and associate professor at the School of Speech-Language Pathology and Audiology of the University of Montreal, who trained these students to provide safe content for the participants. Sessions are always preceded by a few minutes of informal verbal exchange between the facilitator and the participants. When the session starts, the facilitator briefly presents the session's content and course and then, the participants' microphones are turned off for the exercises. A typical session starts with physical warm-ups consisting of body stretching, soft head, neck, and shoulder movements and breathing exercises to warm up the participants for using their voices, for approximately 5–8 min. Then the session proceeds with vocal exercises that are planned in order of complexity, beginning with simple vowel productions, followed by short syllables, non-words and words, and then sentences and texts (poems, songs, etc.). The facilitator first models the exercise, second the facilitator encourages the participants to repeat it with them, and finally to repeat it on their own. The facilitator regularly invites the participants to be mindful of their own sensory perceptions while doing the exercises, and to explore their own voice in terms of loudness, pitch and articulatory precision and rate, while respecting their own limits and comfort. At the end of the session, the microphones are opened again for ~5 min, and an exchange ensues between the participants and the facilitator during which questions and requests are addressed. Upon the request of the participants, some special sessions also offered educational content regarding vocal production mechanisms, tutorials to better use and benefit from the Zoom technology, and tutorials to navigate the health system to access SLT services.

MdlV is grounded in a partnership approach which entails mutual recognition of expertise between participants and facilitators (11, 12). The partnership is facilitated through the online discussions following the sessions and participants reaching out by email to share their feedback, experience, and advice to the facilitators as well as questions to gain additional knowledge regarding voice (e.g., physiology). This approach is aimed to empower MdlV participants and to improve the planning and design of the sessions to closely fit their needs. During the period of April 2020 to July 2020, there was a mean of 42,29 participants per session, a minimum of 29 participants and a maximum of 71.

The development of MdlV occurred during the explosion of health related innovations that emerged around the globe in response to the COVID-19 pandemic, including solutions related to the recruitment of healthcare providers and volunteers as well as to the redesign of health care delivery *via* telehealth and virtual solutions (13). With regards to individuals living with Parkinson's disease, the increased use and development of web-based exercise programs, apps and online peer support group during that time has opened the discussion regarding the potential relevance of maintaining e-solutions beyond the pandemic (14–16). Indeed, unexpectedly high recruitment rate for Zoom-based physical exercise program have suggested the readiness of individuals living with PD to participate in telerehabilitation (14, 15). Further, the potential of online interventions to foster accessibility of self-management interventions by overcoming some barriers to take part in face-to-face activities (e.g., cost, lack of services in remote areas) has been highlighted (15, 17). In order to clarify which of the multitude of innovations that have been developed in response to the pandemic should be retained when we move forward, it is fundamental to evaluate the impact of these novel solutions on patients (13), as well as reflect on the ethical, legal, social, economic and environmental issues they might raise (18). To this end, the present study aimed to explore participants' experience and perception of MdlV in terms of participation, motivations, feelings, perceived outcomes, and appreciation. The research questions that guided our study were (1) Did this initiative succeed in its goal to overcome social isolation due to the pandemic?; (2) Are there any other benefits to this activity than the social ones?; (3) Is there a place for this online vocal activity within the existing continuum of care and services available to people with PD?

## Methods

### Context

This study is based on data acquired by the organism Parkinson Québec (PQ) as part of a service quality study. This study was approved by the *Centre de Recherche Interdisciplinaire en Réadaptation du Montréal Métropolitain* (CRIR) Ethics Committee (CER 2022-1357) as secondary data analysis.

### Participants

To take part in this study, participants had to be a member of PQ and to have registered to participate in the activity MdlV for Summer 2020. There were no exclusion criteria.

### Data collection

A ten-question survey, deliberately kept short to foster a high response rate, was specifically designed for the purpose of this study by the research team, in collaboration with PQ. The survey included four closed-ended questions about respondents' habits as MdlV participants (e.g., participation rate, duration of participation) and history of SLT services. It also comprised six open-ended questions regarding respondents' experience of SLT services, motivation to take part in MdlV, feelings before and after a MdlV session, perception of changes attributed to MdlV and recommendations to improve the activity. The full survey is presented in the Supplementary Material. On July 20^th^, 2020, PQ sent an email including a link to the survey hosted by Google Form to the 45 participants registered to MdlV. They had 25 days to answer the survey anonymously and voluntarily before it was closed.

### Data analysis

The responses to the four closed-ended questions were analyzed using descriptive statistics (e.g., percentages). The quantitative analysis was performed in Microsoft Excel. Participants' statements provided in response to the six open-ended questions were subjected to an inductive qualitative content analysis. First, CD and EC read all responses to familiarize themselves with the data. Second, they independently coded participants' responses to each question. In case of disagreement, CD and EC discussed and adjusted some codes until they reached consensus. Third, MCH reviewed the coding developed by CD and EC by reading each participant's response as well as the code(s) they were assigned to. In this process, she suggested modifications in the coding by merging similar codes under new categories, by renaming some codes and by creating some new codes. For instance, she grouped the former codes “advice/tools,” and “animation team” under the new code “supporting team,” she renamed the former code “reassuring oneself/not being alone with PD” under the new code “belonging to a group” and assigned both codes under the new category “Seeking peer and professional support.” Modifications suggested by MCH were reviewed by CD, EC and IV who agreed with the changes. Finally, once the team had reached consensus for the codification and categorization of each question, the dataset was looked as a whole. This allowed the team to observe similarities among statements regardless of the question they belonged to. Categories belonging to a different question but pertaining to a similar idea were thus assigned to a same theme. For instance, the categories “improved function and comfort in swallowing or speaking and improved communication skills” and “positive emotional and energy state” were grouped under the same new theme “Unanticipated benefits and desired gains catalyzing motivation to participate in MdlV.” The final dataset on which the whole team agreed includes four themes.

## Results

Among the 45 members of PQ who were invited to complete the survey, 37 members fully answered to the survey (response rate of 82%).

### Quantitative results

The majority of respondents (62,16%) participated to MdlV since the beginning of this activity. In terms of frequency of participation, most respondents (59,46%) participated on a daily basis, i.e., 5 days per week. A vast majority (83,78%) heard about the activity by Parkinson Québec, the organism hosting MdlV, while others heard about it either from their speech-language therapist (10,81%) or from their family and friends (5.41%). Most of the participants (72,97%) had never received SLT services before. Finally, nearly all respondents (97,30%) intended to keep participating in the activity (see Table 1).

**Table 1:** Participants' level of participation in MdlV and history of previous SLT services.

**Variables**	***N***	**%**
*Duration of participation*	37	100.00
For 13 weeks	23	62.16
For at least 3 weeks	8	21.62
For <2 weeks	4	10.81
Never participated or abandoned	2	5.41
*Frequency of participation*	37	100.00
Daily participation	22	59.46
3–4 times per week	12	32.43
Less than twice a week	3	8.11
*How they heard about MdlV*	37	100.00
By Parkinson Québec	31	83.78
By their friends or family	2	5.41
By a speech-language therapist	4	10.81
*History of speech-language therapy services*	37	100.00
Yes	10	27.03
No	27	72.97
*Intent to pursue the activity*	37	100
Yes	36	97.30
No	1	2.70

### Qualitative results

The qualitative analysis yielded four themes: “Previous SLT services are variable and perceived as beneficial but with limitations;” “Seeking improvement and support as initial motivations to engage in MdlV”; “Unanticipated benefits and desired gains catalyzing motivation to participate in MdlV;” and “Perceived limitations of MdlV and persisting needs.” Each theme will be presented in the following paragraphs and tables including their corresponding subthemes, categories, codes and excerpts are provided.

#### Previous SLT services are variable and perceived as beneficial but with limitations

This theme referred to respondents' experience and perception of previous services in SLT and included two subthemes: “Variability of previous SLT services” and “Appreciation of SLT services” (see Table 2). The first subtheme highlighted that participants' description of the SLT services they had received differed from one respondent to another in terms of frequency, intensity, duration, modality, and nature of intervention. For instance, services ranged between 4 weeks and 4 months, could involve either individual or group interventions and entail swallowing assessment, advice or vocal, respiratory and/or facial muscle exercises. The second sub-theme encompassed participants' positive and negative appreciation of SLT services. With regards to the perceived benefits, participants alluded to the efficacy and usefulness of SLT services, their positive impacts on their articulatory and vocal functions as well as on their self-confidence and social participation. For instance, a participant mentioned: “It helped me regain self-confidence to dare expressing myself in groups.” As for the perceived limitations and negative repercussions of previous SLT services, participants reported persisting needs for individual services, a lack of benefits over time, and a sensation of irritation associate with high vocal intensity exercises.

**Table 2:** Previous SLT services are variable and perceived as beneficial but with limitations.

**Sub-theme**	**Category**	**Code**	Excerpt^*^
Variability of previous SLT services	Frequency, intensity, duration, and modality of services	4 week intensive program	“Lee Silverman program.”
		Programs longer than 4 weeks	“6 weeks in small group, exercises to strengthen the voice. Effective, but short-lived effect.”
		Individual speech therapy follow-up for a few sessions	“The speech therapist gave me advice and exercises to do. I saw them three times.”
		Group	“6 weeks in small group […]”
	Nature of the interventions	Swallowing assessment	“Video Fluoroscopy, practice with the EMST-150, projecting high- and low-pitched sounds, reading aloud. The only drawback is that the sounds or reading at high decibel irritates my throat!”
		Advice	“The speech therapist gave me advice and exercises to do […]”
		Vocal, respiratory and/or facial muscle exercises	“It was to help me with my voice problem, and with the exercises it helped me to regain confidence in myself to dare to express myself in group.”
Appreciation of previous SLT services	Perceived benefits	Effectiveness/utility/ positive appraisal	“An hour a day for 4 weeks. Very helpful.”
		Perceived benefits on function (articulation, vocal tone)	“4 week program, 4 h per week. Excellent for pronunciation and tone of voice.”
		Perceived benefits to self-confidence and social participation	“[…] it helped me to regain confidence in myself to dare to express myself in group.”
	Limitations/perceived negative impacts	Persistent need for individual support	“[name of the rehabilitation center] for a month every afternoon. I liked it but I would have needed a follow-up.”
		Limited maintenance of benefits over time	“6 weeks in a small group, exercises to strengthen the voice. Effective, but short-lived effect.”
		Sensation of irritation related to high intensity vocal exercises	“[…] The only drawback is that the sounds or reading at high decibel irritates my throat!”

* *For the sake of concision only one excerpt is provided per code*.

#### Seeking improvement and support as initial motivations to engage in MdlV

This theme involved two subthemes characterizing participants' main motivations to participate in MdlV (see Table 3). First, the “Desire to improve one's communication and swallowing skills in the face of perceived limitations” highlighted that respondents observed a decrease in their swallowing, language and/or speech function as well as experienced communication breakdowns and, as such, they wanted to improve these functions and increase communication successes. For instance, a participant referred to the “need to be understood and not repeat” as a main reason to take part in MdlV. Second, “Seeking peer and professional support” evoked participants' desire to obtain support by belonging to a group and by the guidance of a team. The following excerpt exemplifies one participant's need for peer support: “to notice that I'm not alone. Alone with a voice problem caused by Parkinson's.”

**Table 3:** Seeking improvement and support as initial motivations to engage in MdlV.

**Sub-theme**	**Category**	**Code**	Excerpt^*^
Desire to improve one's communication and swallowing skills in the face of perceived limitations	Perceived decreased function and/or desire to improve function with regards to swallowing, language or speech and its dimensions	Swallowing	“To improve my swallowing and my speech...”
		Language	“Voice too weak. Difficulty expressing myself in a group, collecting my ideas to present them.”
		Breathing	“Improve my voice, flow and breath.”
		Voice (general, intensity, quality, tone)	“My voice was hoarse, and I couldn't talk for long. I got tired very quickly and people had difficulty understanding me.”
		Articulation	“Pronunciation practice.”
		Speech rate and fluency	“My voice was completely off, and I was stuttering (palilali). I still stutter, but my voice is much stronger now.”
		Observation of improvement	“My voice was hoarse, and I couldn't talk for long. I got tired very quickly and people had difficulty understanding me. My voice has improved a lot, people tell me spontaneously and I speak more often, and I am more sociable!”
	Disability related to communication and/or desire for communication success	Doesn't make oneself understood	“My voice is fading more and more and I'm not articulating as well, so I'm being asked to repeat myself more and more often.”
		Desire to be understood	“Need to be understood and not to repeat.”
Seeking peer and professional support		Belonging to a group	“First of all your precious advice, and also to see that I am not the only one with a voice problem caused by Parkinson's.”
		Supporting team	“To improve my vocal process and to have an assiduous reference group and creative, dedicated, and passionate facilitators.”

* *For the sake of concision only one excerpt is provided per code*.

#### Unanticipated benefits and desired gains catalyzing motivation to participate in MdlV

This theme referred to the benefits, anticipated or not, that the participants attributed to their participation in MdlV and which seemed to transpose their general initial motivation into positive anticipation before each session. It included three subthemes: “Unanticipated benefits,” “Desired gains” and “Looking forward to MdlV sessions” (see Table 4). The first subtheme pertained to the benefits that participants noticed during or after a session of MdlV. Such positive and immediate consequences to their participation were not necessarily what participants had expected to obtain from this activity in the first place, yet they seemed appreciated. These benefits included a positive state in terms of emotions and energy, with participants reporting increased levels of energy and feeling calm, satisfied, proud or motivated as well as improvement of one's focus. The second subtheme regarded participants' perception of gains that were aligned with their initial desire for support and improvement in communication and swallowing. It encompassed five categories referring to: an improvement in the function and comfort of swallowing or speech as well as in communication skills; an awareness of oneself, of the vocal and swallowing processes and of strategies fostering these processes; confidence in one's abilities and in the possibility of improving; satisfaction with the team of facilitators; and satisfaction with the group. The third subtheme referred to participants' positive anticipation before a MdlV session. A vast range of feelings were evoked including confidence, motivation, interest, excitement, satisfaction and contentment as illustrated in the following excerpt: “I'm always looking forward to meet with the group.”

**Table 4:** Unanticipated benefits and desired gains catalyzing motivation to participate in MdlV.

**Sub-theme**	**Category**	**Code**	Excerpt^*^
Unanticipated benefits	Positive emotional and energy state	Good energy level	“I am often very satisfied with the exercises, my energy level is good.”
		Pride/feeling of deserving a reward	“Good, and happy with myself.”
		Motivated/stimulated	“I am very happy with the session and I am stimulated; I always find that it goes by too fast...”
		State of calm/quietness	“I feel calm and look forward to the next day's class […].”
		Satisfaction	“Satisfaction of participating.”
	Improvement of the physical and mental state	Better concentration	“Giggles and better concentration and energy.”
		Improvement of the energy level	“I am happy and my energy level is better.”
Desired gains	Improved function and comfort in swallowing or speaking and improved communication skills	General improvement	“Yes, and I've even had favorable comments from friends to that effect.”
		Swallowing	“My swallowing is better, I force my voice less, less irritation.”
		Breathing	“Difficult to evaluate. Improved breathing technique by filling our lungs more with air and trying to control the exhalation.”
		Voice	“Better than at the beginning of the semester, my voice is also better.”
		Articulation	“Yes better pronunciation due to the guidance provided.”
		Tone	“I'm sure it does. It helps me to put intonation and emotion.”
		Speech rate and fluency	“I stutter when I am stressed or very tired, but less than before. I manage to have “almost” normal conversations.”
		Successful communication in everyday life	“Certainly, in each of the areas you listed, including voice, speech, swallowing, and my communication habits such as meeting people and answering the phone.”
		Reduction of effort and discomfort	“My voice breaks less and I force it less.”
	Awareness of self, of the process underlying voice production and swallowing and of strategies supporting these processes	Awareness of one's voice and strategies to improve it	“Yes, I am more aware of the importance of making the necessary efforts to express myself clearly and I am more confident that by working on it I will improve my condition.”
		Awareness of the process of speech production and its complexity	“I am more aware of the complexity of the voice.”
		Awareness of the swallowing process	“I am more aware of what comes into play when I speak and swallow and it helps me to be understood better.”
	Confidence in one's abilities and in the possibility of improvement	In one's abilities	“Above all, more confidence and assurance for daily communication.”
		In the possibility of improvement	“Yes, I am more aware of the importance of making the necessary efforts to express myself clearly and I am more confident that by working on it I will improve my condition.”
	Satisfaction with the team of facilitators	Appreciation of the variety among facilitators	“At the moment I am very satisfied, which may depend on the fact that we have a new facilitator every day where each one brings something different to the table.”
		Appreciation of the facilitators	“[…] The facilitators are good: we all enjoyed them.”
	Satisfaction with the group	Sense of belonging	“Always happy with the course and in a good mood with our beautiful gang.”
Looking forward to MdlV sessions	Positive emotions felt prior to the session?	Motivation	“I am motivated, I like these sessions.”
		Positive anticipation	“I enthusiastically anticipate sessions and enjoy the group dynamic […].”
		Satisfaction before the start of the session	“Simply happy to be there and energized by the anticipated pleasure.”

* *For the sake of concision only one excerpt is provided per code*.

#### Perceived limitations of MdlV and persisting needs

This theme alluded to the limitations of the activity, as perceived by participants, as well as to the individual needs that could persist despite taking part in this activity, as revealed through participants' suggestions to improve MdlV. It comprised four sub-themes: “Suggestions to meet individual needs,” “Non-optimal state of predisposition to the activity related to schedule and modality,” “Mixed perception regarding one's improvement as a result of the activity” and “Negatively perceived state following the session” (see Table 5). The first subtheme included every suggestion that participants made for the activity to be more personalized to their own needs and situation. These recommendations were varied and pertained to different aspects of the activity, namely the type of exercises, how the session works, planning and follow-up of sessions, and schedule and length. The second subtheme regarded the low energy level and negative emotions felt by some participants before the session. The third subtheme related to either ambiguous improvement or lack of improvement as a result of one's participation to MdlV and included statements such as: “[…] My husband has observed a better speech and better communication whereas I don't observe that much improvement. […].” Finally, the fourth subtheme pertained to participants' negative emotion and state immediately after the session including deception, low energy and throat irritation.

**Table 5:** Perceived limitations of MdlV and persisting needs.

**Sub-theme**	**Category**	**Code**	Excerpt ^*^
Suggestions to meet individual needs	Type of exercises	Maintain or have more exercises in the context of song, read aloud and theater excerpts	“I am searching for my words, which makes it difficult to have a conversation. I wish we could continue with the songs because it helps me a lot.”
		Proposal and assessment of vocal exercises	“More exercises with projected voice.”
		Add breathing exercises	“Do not hesitate to include articulation and breathing exercises. But the current exercises are fine. If more time was available – for example, 3/4 of an h - that would be good.”
		Direct and indirect request to add articulation exercises	“My articulation is blurred and my pronunciation of some words is difficult.”
		Suggestion to address word retrieval difficulties	“More time to practice readings-songs-intonations through theater excerpts and talk about other disorders like forgetting the path to words and saying a word when you want to say another one and not realizing it right away...”
		More exercise and less warm-up	“Do the voice exercises longer. Perhaps reduce the warm-up period at the beginning and focus on the voice.”
		Maintain variety in the exercises	“Please continue to encourage variety in the sessions. Thank you so much! You are appreciated.”
	How the session works	Record Zoom sessions to allow asynchronous access	“I can't always attend the sessions and would like to be able to pick up the missed sessions later. These should be recorded and made available to participants.”
		Get individual feedback	“Difficult to do but it would be very beneficial if the facilitators could hear us individually and suggest corrections or ways to go beyond the individual practice without feedback.”
		Include a question-and-answer period	“A place where one could ask questions without disrupting the meeting.”
	Planning and follow-up of sessions	Reduce, remove, or substitute homework with optional exercises	“Provide access to a series of free exercises on Facebook, rather than talking about homework.”
		Know the content of the sessions in advance	“[…] Is it possible to have the schedule of each of you, maybe some techniques would be more beneficial to each of you if you knew in advance. Thank you for what you do, it's great.”
	Schedule and length	Change the time of the sessions (earlier) and their frequency (reduce the number of times per week)	“I would like the activity to be given at another time of the day (e.g., 11 am or 4 pm) away from lunch time. But even at the same time, I would be very happy to still participate in this activity.”
		Increase the length of the sessions	“Add 15 min to practice our voice, session too short.”
		Maintain schedule and duration	“Keep the same schedule and duration.”
Non-optimal state of predisposition to the activity related to schedule and modality	Low energy level	Fatigue/low to very low energy level	“[…] My energy is often low at the end of the day.”
	Negative emotions felt	Nervousness/stress/negative anticipation	“The first few sessions a little anxious (connection problem!!) if not I always look forward to meeting the group.”
		Sadness	“Sadness and little energy.”
Mixed perception regarding one's improvement as a result of the activity	Lack of improvement or ambiguous improvements	General comment	“No [I didn't observe changes].”
		Optimism despite lack of improvement	“No, because I just started. But I'm hoping that “When I start again this fall, there will be some positive changes that will happen […].”
		Attribution of lack of improvement to lack of participation	“I did not participate enough.”
		Ambiguous improvements	“Yes, a lot of improvement. However, I started taking medication at the same time. Is it one or the other?...Both matters to me...”
Negatively perceived state following the session		Deception	“Deception. […]”
		Fatigue/low energy	“ […] Decreased energy.”
		Throat irritation	“I'm fine ... sometimes a little irritation of the throat ... glad to have participated.”

* *For the sake of concision only one excerpt is provided per code*.

## Discussion

Through the use of a survey exploring participants' experience and perception of MdlV in terms of their participation, motivations, feelings, perceived changes and appreciation, we intended to evaluate the impact of the crisis-led innovation, MdlV, on individuals living with PD. Our quantitative results suggest that this activity was appreciated by its participants as the majority of respondents not only took part in MdlV on a daily basis since its beginning, but were also interested in maintaining their participation in the future. Our results also highlighted that most respondents had not previously received SLT services and that they heard of MdlV through Parkinson Québec. Our qualitative analysis allowed to further expand and explain these quantitative results. The first theme “Previous SLT services are variable and perceived as beneficial but with limitations” expanded on the nature and impacts of SLT services previously received by a minority of participants. The two following themes shed light on the factors that may explain participants' high level of engagement and interest in MdlV: “Seeking improvement and support as initial motivations to engage in MdlV” and “Unanticipated benefits and desired gains catalyzing motivation to participate in MdlV.” The last theme nuanced that MdlV could not meet all participant's needs: “Perceived limitations of MdlV and persisting needs.” In the following paragraphs, we will frame our discussion of these results and their clinical and research implications around the three research questions that guided this study.

### Did MdlV succeed in its goal to overcome social isolation due to the pandemic?

Seeking peer and professional support was identified as one of the main motivations to take part in MdlV, which is also consistent with one of the purposes of MdlV, namely to break social isolation of individuals with PD. Our results suggest that participants' need to belong to a group and to receive support from professionals was fulfilled as they reported satisfaction toward the group of participants and the team of facilitators. In addition to overcoming social isolation, the group format of MdlV may also have fostered factors and strategies documented as key in the wellbeing of individuals with PD, including to be thankful for one's current situation by comparing to others in worse situations, integrating humor in everyday life, relating to and understanding the experiences of others diagnosed with PD, and positive interactions with health professionals (4). Such results are consistent with previous studies which have documented the positive impact of community programs for people with Parkinson's disease especially when the program is group-based and group-supportive (3). Indeed, programs that focused on group supportiveness and group exercises have been proven to have a positive impact on wellbeing, to reduce anxiety and depression, and to reduce social phobia (3).

### Are there any other benefits to MdlV than the social ones?

Our results suggest that one of the two main motivations for participants to take part in MdlV is the desire to improve their language, speech and swallowing functions and their communication. Participants reported benefits related to these skills, such as improvements in speech and its components (e.g., voice, breathing, articulation), and increased communication success and confidence in their communication skills. Communication disorders in the context of PD contribute to decreased self-confidence and engagement in social activities which can then lead to social anxiety and isolation (4). In a context where the social isolation that can characterize the experience of PD in general may have been exacerbated by the pandemic, it is not surprising that a desire to act on one of the factors that can impede social interactions was a central motivation for taking part in the MdlV activity. Moreover, this motivation of the participants is consistent with the dual purpose of MldV at its inception, namely to break isolation through voice stimulation activities in order to continue using the vocal apparatus, which was at risk of being under-stimulated by the lack of interaction generated by social distancing measures. The communicative gains perceived by the participants may thus have acted as a lever to break isolation and promote greater social participation in general.

Participants also noted being more aware of themselves, of the process underlying voice and swallowing as well as of the strategies supporting these processes. Such outcomes, that respondents attributed to their participation in MdlV, are coherent with their initial desire to improve their speech and swallowing functions and communication. In fact, the combination of increased knowledge and heightened confidence in one's abilities may represent the mechanism underpinning participants' perceived gains. Patient education (e.g., through explanations of voice production and modeling of exercises) may have increased participants' knowledge, improved their self-efficacy and fostered their uptake of behaviors (e.g., practicing speech exercises, engaging in conversations) (19, 20) conducive of improved speech function and communication. In addition, a raised awareness of oneself and increased knowledge may have supported participants' self-management of their symptoms (11, 12) and promoted their wellbeing (4).

Unanticipated benefits, i.e., neither expected by the team who designed MdlV, nor by the individuals who decided to engage in this activity, were reported by the participants. Such benefits, which consisted of a positive mood at the end of the sessions could also have contributed to participants' adaptation to PD related changes and symptoms. For instance, the state of calmness, tranquility, and satisfaction experienced by participants after the sessions seems consistent with the adoption of a more harmonious, present-moment focused state of mind that is a cognitive strategy that may influence the wellbeing of individuals with PD (4). Such a state of wellbeing at the end of the sessions could moreover be explained by the following physical strategies promoted by MdlV activity, namely controlling one's movements and actions by taking one's time and performing exercises mindfully (4). Furthermore, motivation and belief in their own abilities are two internal states required to cope with the difficulties caused by PD symptoms (5) that may have supported the coping mechanisms of participants in MdlV who reported feeling motivated at the end of the sessions and believing more in themselves, in the possibility of improvement, or in their ability to communicate despite imperfect speech. This mechanism can be related to the experience of hope, a sentiment that plays a key role in the process of care and resilience in chronic illness (21).

Thus, MdlV appears to have benefits beyond the virtual social meeting space created during the moment of participation in the daily sessions. The activity seems to support a virtuous loop of empowerment of the participants where the nature and modality of the proposed exercises reinforce their awareness, knowledge, skills and/or their belief in their skills, thus motivating them to engage in social situations outside of MdlV, generating new opportunities to use and maintain their communicative abilities.

Although MdlV seems to have brought both social and communicational benefits to the participants, it may nonetheless have left some participants with unfulfilled needs (e.g., to obtain personalized feedback), a suboptimal state following a session (e.g., feeling disappointed), or the impression that their participation did not bring positive changes. This group activity may therefore not allow to meet the needs of the diversity of profiles of individuals living with PD who may differ with regards to the stage of the disease, the type and severity of symptoms and their level of acceptance of the diagnosis. Unmet needs related to individual speech or voice deficits does not come as a surprise considering the general nature of the stimulation exercises that are provided. It is unrealistic and also not the objective of MdlV to provide individual therapy advice. Regarding level of acceptance, people who reject to identify themselves by their disease to avoid the stereotypes associated with it (4) may therefore not want to take part in this group or refuse to be exposed to others' progression of the disease (3). On the contrary, those who identify themselves by their diagnosis and relate to the new lifestyle it has entailed may more actively seek to interact with other individuals living with PD (4).

In a future study it would thus be important to better elucidate what aspect of MdlV works for whom, in which context, and how. Although MdlV includes many components of other interventions that have been previously evaluated, it constitutes an innovation because the combination of these components is unique. For instance, while MdlV can be likened to online support group in that participants meet synchronously and virtually for several weeks with the opportunity to chat during the first few minutes of the session, it is not a support group *per se*, as it does not focus on the expression of thoughts and emotions and on receiving and offering support (22). It can also be similar to a face-to-face group therapeutic singing (23, 24), but delivered virtually and without auditory feedback of other participants, or to the online viewing of a pre-recorded group therapeutic singing, but by being synchronously present with other participants (25). Furthermore, MdlV can resemble in some ways the remote delivery of Lee Silverman Voice Treatment as vocal and respiratory exercises are virtually delivered up to four times a week for several weeks, but differ as it is not delivered one-on-one by a SLT who can provide feedback (26–28). Since MdlV was developed rapidly in response to an urgent need triggered by the pandemic, the explanatory nature of the present study does not allow for documentation of its effects on measures (e.g., quality of life, stigma, social support, respiratory pressure, sound pressure) previously used to evaluate similar interventions (22, 23, 25, 28) nor does it allow for targeting which component(s) of this activity are critical. However, the unique nature of MdlV as well as the positive results of the present study in terms of participants' interest and perceived benefits warrant future studies seeking to clarify the mechanisms of action and effects of this intervention. A future realist evaluation using data from internal documents related to the development and implementation of this activity, from the perspective of stakeholders (e.g., participants, facilitators) and from peer-reviewed studies of similar interventions, would allow to generate an explanatory theory of this innovation which could be further tested (29–32).

### Is there a place for MdlV within the existing continuum of care and services available to people with PD?

Our results show that the majority of respondents (73%) had never received SLT services. This statistic is consistent with what other previous studies reporting access rate to SLT services between 37 and 59% (33–35). Even though SLT interventions exist that can help maintain speech and vocal function and mitigate the negative effects of their decline (36, 37), barriers of multiple nature, which complicate access to SLT services have been documented in Canada (38) and worldwide (33, 39). These barriers include mobility issues related to reduced motor ability or complicated logistics, costs, and lack of awareness of the role of SLTs among both patients and medical staff (33). There can be significant delays between the time patients receive their diagnosis and their first SLT treatment and the treatment does not necessarily take place at the time in the patient's life when exercises are applicable or necessary for them (33, 40). Although MdlV was not designed as a therapeutic service, it was crafted by students and a researcher in SLT building on principles of neuroplasticity (10) and vocal function exercises (41). Participants report improvements in their communication skills that they link to their participation in MdlV and feeling empowered by the tools they are being proposed with at MdlV, such as “enthusiasm regarding the possibility of learning and improving despite occasional setbacks” and “mostly [having] more confidence and self-assurance for everyday communication.”

These positive results suggest that independently of the COVID-19 context, MdlV could have an inherent value as a non-therapeutic service supporting communication abilities by providing general information on speech and vocal function that supports functional improvement of specific speech components and self-management, through increased knowledge, self-awareness, motivation and confidence to engage in social situations requiring speech. This kind of service appears as important in today's health system's landscape considering not only the restrained access to SLT services and that the long-term benefits of SLT interventions are limited (42), but also the projected increase of the older population and associated neurological conditions such as PD (43), that will inevitably increase the demand for services and amplify accessibility issues.

Accessibility issues are indeed raised as a major health system challenge worldwide and low-cost community-based innovations that require little training on the part of the service providers are advanced as responsible solutions that should be prioritized (44). MdlV was designed as a service requiring entry level expertise by the direct provider, indeed the sessions are not given by SLTs directly but by undergraduate and graduate students who were given a short training as facilitators by SLTs. Moreover, the activity is distributed by a community partner in virtual format. This model ensures low cost (the activity is free for members of Parkinson Québec), by decreasing financial barriers to accessing the service, and permitting to reach a large group of geographically separated people simultaneously, removing the mobility barriers. Nevertheless, although more seniors are incorporating technology into their daily lives (45), it should not be overlooked that offering this online nature of MdlV can also add barriers to persons who do not have access to the necessary technology. It would thus be important to think about services that simultaneously incorporate an online component and a face-to-face one. Moreover, the fact that MdlV is made available through a community partner circumscribes its accessibility to the partner's members only. At the same time, this facilitates access to the activity by disseminating it to a large pool of target individuals, who otherwise might not have heard of voice and speech related interventions through their physician, and reducing individual costs. Indeed, most of our respondents (83.78%) had heard of MdlV through our community partner Parkinson Québec. In the future, building on partnerships with diverse associations or institutions may leverage the expansion of MdlV or the development of similar activities targeting the voice and speech symptoms of individuals with PD.

Knowing that a lack of awareness in both patients and medical staff regarding existing SLT services has been identified as a barrier to access (33, 39), MdlV's potential as a platform to empower patients to ask for these services could also be seen as a lever to increase access by driving a bottom-up awareness in the medical staff. In that sense, individuals experiencing that MdlV is not meeting their needs in terms of speech or voice, that we lifted as a negative outcome of MdlV in the above section, might in fact be valuable if it motivates participants to seek out additional help. On the positive side, the general nature of MdlV also contributes to its accessibility by situating it as an inclusive actor in the continuum of care by offering an exercise space used by both participants that have not yet accessed specialized care and participants that are seeking support for self-management and self-care after their rehabilitation services ended. A realist evaluation, such as suggested above, could also help elucidate how participants with diverse profiles regarding the severity and nature of their symptoms make use of MdlV.

Finally, having in mind that our participants reported raised awareness and knowledge attributed to their participation in the activities, MdlV could have a value beyond the context of the pandemic as an educational service. Education around speech symptoms and their management could not only benefit patients directly but also their spouses or caregivers. Indeed, both patients with PD and their caregivers find it difficult to identify symptoms and understand the disease (46). It is not surprising, therefore, that several of the participants at MdlV actually had their spouses with them or around them during the sessions. Thus, given that improved understanding of the disease might reduce the burden of care both for patients and caregivers (47), and given the role of communication in perceived burden of care (48), future studies could also address the outcomes of MdlV in both participants with PD and their spouses or other caregivers.

### Strengths and limitations

For our study, we had high response rates as well as a wide variety of respondent profiles. However, we must consider a selection bias in the responses since perhaps only the most motivated of the group would have responded. In addition, the survey used in this study was not constructed from a scientific point, since it was designed rapidly in response to a spontaneous request by our community collaborator. We also lack data on the profile of the respondents (for example: for how long they have had PD, or their racial, cultural or gender identity) that could have provided us more insight into whom the activity is accessible for and benefits. For instance, it could have helped clarify how the profile of MdlV participants is similar or not to that of Parkinson Québec members in general. Such information would also have facilitated the comparison of our results with the existing literature addressing voice rehabilitation and community services for persons with PD as well as the crafting of recommendations for improvement. Finally, although we gathered information related to participants' previous experience of SLT services, we did not ask those who had no previous experience of SLT the reasons explaining this situation. This would have contributed to better situate the relative advantage and/or appeal of this online vocal stimulation group activity over existing SLT services. To address these limitations, future studies examining the efficacy and mechanisms of MdlV should gather information regarding individuals who participate in MdlV and those who decided not to register, including with regards to their previous experience or lack of experience of SLT services.

## Conclusion

In conclusion, MdlV constitutes an innovative activity embedding several components of interventions that have been previously delivered and studied, but not in the form proposed here, i.e., large group online delivery of vocal exercises modeled and encouraged by a facilitator who has no auditory feedback of participants' performance. Our study of participants' experience and perception of MdlV highlighted the high level of engagement and interest of participants as indicated by a high response rate and a strong majority of respondents expressing a desire to maintain their participation in the future. Such a motivation toward this activity could be explained by a combination of participants self-reported gains, which were aligned with their initial motivations to improve their communication and to obtain support, and unexpected yet valued benefits on their emotional, physical, and mental state. Taken together, MdlV thus seems to have reached its goal of overcoming social isolation during COVID-19 as well as entrained additional benefits such as perceived improvement in communication, confidence and self-awareness. MdlV could be seen as a platform that SLTs can deploy in collaboration with community partners to make voice and speech stimulation accessible to a large public in parallel to promoting the role of SLTs and existing services for persons with PD. However, future studies should nevertheless clarify with stakeholders how to embed such a service efficiently and responsibly within the continuum of services delivered to individuals living with PD. Furthermore, MdlV did not seem to meet all participants' needs as some formulated recommendations to improve the activity. Given these limitations and considering the innovative nature of MdlV, future studies should also address what different profiles of participants benefit from this activity and through which mechanisms.

## Data availability statement

The data analyzed in this study is subject to the following licenses/restrictions: The data that was used in our study was collected by a survey that our community partner (Parkinson Québec) distributed to participants in the activity that this project is studying. Anonymized data-set was provided to the researchers by Parkinson Québec and the Ethics Committee deemed this study to be “secondary use of data”. We do not have the partner's consent to make the data set public. Requests to access these datasets should be directed to ingrid.verduyckt@umontreal.ca.

## Ethics statement

The studies involving human participants were reviewed and approved by Centre de Recherche Interdisciplinaire en Réadaptation (CRIR) du Montréal Métropolitain. Written informed consent for participation was not required for this study in accordance with the national legislation and the institutional requirements.

## Author contributions

The survey was codesigned by IV and collaborators at Parkinson Québec and MdlV. CD analyzed the quantitative data, while M-CH, CD, ÉC, and IV analyzed the qualitative data. All authors have contributed to the preparation of the manuscript, read, and approved it.

## Funding

This study was supported by Parkinson Québec and by the Ministry of Economy and Innovation of the province of Quebec, Canada [Grant number 20-23-PSOv2a-51656].

## Conflict of interest

Author IV was a co-founder of MdlV and of the non for profit that was later created to insure its continuity, she has also acted as a facilitator in its beginning. CD, ÉC, and OR have been acting as facilitators for MdlV, but not during the period that this study is reporting on. The remaining author declares that the research was conducted in the absence of any commercial or financial relationships that could be construed as a potential conflict of interest.

## Publisher's note

All claims expressed in this article are solely those of the authors and do not necessarily represent those of their affiliated organizations, or those of the publisher, the editors and the reviewers. Any product that may be evaluated in this article, or claim that may be made by its manufacturer, is not guaranteed or endorsed by the publisher.
