# Supplementary material for: Participants' perspective on a COVID-19 online vocal group stimulation for people with Parkinson's disease
Source: Front Rehabil Sci. 2022 Aug 2;3:951426. doi: 10.3389/fresc.2022.951426 (PMC9397883; doi:10.3389/fresc.2022.951426)
Supplement: Supplementary file 1 [file Table_1.DOCX]

Supplementary Material

**Survey Musculation de la voix**

Thank you for agreeing to answer this survey. It contains 10 questions. Some questions require a free text response. If you don't want to leave a comment, you can just write "no" or any other letter or word and move on to the next question. If you have any questions about the survey, you can reach Ingrid Verduyckt, head of development of Musculation de la voix activity by email: ingrid.verduyckt@umontreal.ca

1. How long have you been participating in Musculation de la voix?

- Since the beginning of the activity, April 20, 2020
- For three (3) weeks or more
- For two (2) weeks or less
- Other (please specify)

1. How many times a week do you take part in the Musculation de la voix activity?

- Daily
- Three (3) days a week or more
- Two (2) days a week or less

1. Where did you hear about this activity?

- Parkinson Québec
- Family or friend
- Other (please specify)

1. Before participating in Musculation de la voix activity, had you received speech therapy services?

- Yes
- No

1. If you want, can you describe the services you have already received and what you thought of them?
2. What are the main reasons that motivate you to participate in Musculation de la voix?
3. Before the session begins, what emotions do you usually feel, what is your energy level, ...?
4. After the session, what emotions do you usually feel, what is your energy level,...?
5. Have you observed any changes in your voice, swallowing, speaking, or communication habits that you believe are related to your participation in the activity?
6. Would you like to continue the Musculation de la voix activities in the fall (starting in September 2020)?
7. Do you have anything to suggest to us so that the activity is better suited to your needs?
